# Global Perspectives on Radiotherapy Utilization for Pancreatic Ductal Adenocarcinoma

**DOI:** 10.1007/s12029-026-01600-0

**Published:** 2026-09-25

**Authors:** Bradley N. Reames, Eric D. Miller, Ziyi Zhou, Amol Narang, Patrick L. Quinn, Bradley White, Emile Gogineni, Aslam Ejaz

**Affiliations:** 1 Department of Surgery, Wake Forest University School of Medicine, Winston-Salem, NC USA; 2 Department of Surgery, University of Illinois Chicago, 840 S. Wood Street, CSB 618, Chicago, IL 60613 USA; 3 Department of Radiation Oncology, Ohio State University, Columbus, OH USA; 4 Department of Radiation Oncology, Johns Hopkins Hospital, Baltimore, MD USA; 5 Department of Surgery, Ohio State University, Columbus, OH USA

**Keywords:** Pancreatic ductal adenocarcinoma, Radiotherapy, Neoadjuvant therapy, Adjuvant therapy, Practice patterns, Multi-modality treatment

## Abstract

**Purpose:**

Pancreatic ductal adenocarcinoma (PDAC) is an aggressive malignancy requiring multi-modality treatment. Radiotherapy (RT) may improve local control and resection rates, though evidence to support use across various stages of disease is mixed and evolving. This study sought to investigate international practice patterns and provider preferences for the use of radiotherapy in the multidisciplinary management of PDAC.

**Methods:**

A 39-item electronic survey was distributed in 2025 through three sub-specialty organizations to providers who have recently treated PDAC.

**Results:**

A total of 176 PDAC providers from six continents completed the survey, with the majority practicing in Europe (*n*= 76, 43%) and North America (*n*= 62, 35%). Respondents included mostly surgeons (*n*= 143, 81%) with 81% (*n*= 142) practicing in academic centers. Nearly 1 in 5 respondents did not offer or consider any indication for neoadjuvant (*n*= 32, 19%) or adjuvant (*n*= 46, 30%) RT. The most commonly cited indication for neoadjuvant RT was for locally advanced disease (*n*= 88, 52%) and surgically unresectable disease (*n*= 86, 51%), while the use of adjuvant RT was primarily for positive surgical margins (*n*= 97, 63%) and positive nodal disease (*n*= 46, 30%).

**Conclusion:**

In an international cohort of surgeons and radiation oncologists, there is substantial variation in perceptions of effectiveness, management preferences, and the propensity to utilize radiotherapy for PDAC. These results underscore the lack of strong evidence to support management, and the need for consensus guidelines and clinical trials to inform best practice.

## Introduction

The role of radiotherapy (RT) for pancreatic ductal adenocarcinoma (PDAC) remains controversial. Following resection, adjuvant radiotherapy may be considered to reduce locoregional recurrence, but data from trials to date have been mixed [1–3]. In setting of localized disease amenable to resection, pre-operative radiation has been explored to improve likelihood of margin negative resection and locoregional control, but randomized data remain conflicting. The PREOPANC trial demonstrated significant improvement in locoregional control with pre-operative chemoradiation compared to upfront surgery in patients with resectable or borderline resectable pancreatic cancer (BRPC), whereas in the A021501 trial, BRPC patients in the pre-operative radiation arm experienced poor outcomes, rendering the role of pre-operative radiation unclear [4, 5]. In the setting of locally advanced pancreatic cancer (LAPC) that is unresectable, consolidative radiation/chemoradiation following chemotherapy has been considered to prevent morbidity/mortality from uncontrolled local progression, but the oncologic benefit remains uncertain [6]. More recently, the role of neoadjuvant radiotherapy for LAPC has been explored, although emerging data is limited, while the role of radiotherapy in patients with metastatic disease remains an area of investigation [7, 8].

As a result, the role of RT in modern multidisciplinary management of PDAC is debated, and current consensus guidelines are inconsistent. Recent guidelines from the European Society of Medical Oncology (ESMO) suggest RT may be considered in the neoadjuvant setting for BRPC following systemic therapy, but is not recommended for LAPC, or in the adjuvant setting [9]. In contrast, guidelines from the American Society of Clinical Oncology (ASCO) suggest CRT may be considered in the neoadjuvant setting for select patients with LAPC after systemic therapy, and in the adjuvant setting for therapy-naïve patients with positive margins (R1) or nodes (N1) [10]. Similarly, guidelines from the National Comprehensive Cancer Network (NCCN) suggest RT may be considered for select clinical circumstances in the neoadjuvant and adjuvant settings, following a robust multidisciplinary discussion [11].

In this context, this study sought to investigate current international attitudes and practice patterns regarding the use of RT in multidisciplinary management of PDAC. To do so, a detailed survey was distributed by email to an international cohort of clinicians treating pancreatic cancer, including surgeons, radiation oncologists, and medical oncologists. Survey domains included practice characteristics, perceptions of effectiveness, and management preferences for RT across clinical stages of PDAC. The primary aim of this study was to characterize current international attitudes and preferences for RT use in PDAC, identifying areas of practice heterogeneity across the multidisciplinary care of these patients. Additional aims were to identify perceived gaps in evidence, inform future research and clinical trials, and potentially improve the care of patients with PDAC.

## Materials & Methods

### Study Design and Participants

An electronic survey was distributed by email and administered through a web-based survey platform (Qualtrics, Provo, UT) between June and August 2025. Surveys were distributed within multiple international sub-specialty societies, including the Radiosurgery Society, International Hepato-Pancreato-Biliary Association (IHPBA), and the Pancreas Club. As a result of this distribution process, the exact number of emails sent, and survey invitations read is unknown. Participants were informed that the survey required 15–20 min to complete and no compensation was offered for participation. A single reminder email was sent to potential respondents one month following the initial invitation. The survey was designed to reach clinicians actively treating PDAC patients across the multidisciplinary team, including surgeons, radiation oncologists, and medical oncologists. An exploratory analysis was performed to identify differences in perspectives between surgical and radiation oncology specialties. To complete the survey, participants first confirmed they had treated patients with PDAC within five years. Respondents who have not treated a patient with PDAC within the last 5 years were excluded from participation.

### Survey Creation and Content

A pilot survey was developed using previously published tools of similar scope and content [12, 13]. Items were modified to fit the topic of RT in PDAC, and a draft was created with input from multiple coauthors. The survey was tested on a local cohort of surgeons and radiation oncologists, and the content, prose, and flow were iteratively refined over six months. Clinicians who participated in survey creation and refinement were excluded from the final study.

The survey consisted of five sections. The first explored practice characteristics including geographic location, years in practice, specialty type, clinical setting, and annual volumes. The second investigated perceptions of effectiveness and the propensity to use neoadjuvant RT across classifications of resectability, while the third surveyed preferences for RT delivery. The fourth and fifth sections evaluated preferences for use of RT in the adjuvant and metastatic settings, respectively. The survey was designed to minimize survey fatigue and maximize meaningful responses. While most items allowed one answer, select items allowed participants to “check all that apply”, and multiple questions provided an opportunity for free responses. The full survey is available in the Appendix.

### Statistical Analysis

Descriptive statistics are presented as number (percent) for categorical data. Missing data, when present, is included in the analysis, resulting in some values that equal less than 100%. For all statistical tests, P-values are two-tailed, and alpha is set at 0.05. All analyses were performed using R version 4.5.3. This study was approved by the University of Illinois Chicago Institutional Review Board.

## Results

### Characteristics of Respondents

A total of 176 surveys were completed. Surgeons completed 143 (81%), radiation oncologists completed 23 (13%), and other PDAC specialists completed 10 (6%) (Table 1). Most respondents were from Europe (*n* = 76, 43%) and North America (*n* = 62, 35%) with the remaining from Asia (*n* = 21, 12%), South America (*n* = 9, 5%), Africa (*n* = 6, 3%), and Australia (*n* = 2, 1%). There was a wide range of post-training experience ranging from 0 to 5 years (*n* = 39, 22%) to over 20 years (*n* = 59, 34%). Most respondents work in an academic teaching hospital (*n* = 142, 81%), with over half (*n* = 90, 51%) practicing at high-volume institutions managing more than 100 PDAC patients annually. Nearly all respondents (*n* = 164, 94%) reported that treatment plans are routinely discussed by a multidisciplinary tumor board. Of note, radiation oncology was represented on these tumor boards in 82% (*n* = 131) of cases, while approximately 1 in 10 respondents (*n* = 19, 11%) did not offer RT at their institution.

**Table 1:** Characteristics of survey respondents

Respondent characteristics	Surgeons	Radiation oncologists	Total cohort^a^
	*N* (%)	*N* (%)	*N* (%)
Practice Region	*n* = 143	*n* = 23	*n* = 176
North America	49 (34)	10 (44)	62 (35)
South America	6 (4)	1 (4)	9 (5)
Europe	62 (44)	11 (48)	76 (43)
Asia	19 (13)	1 (4)	21 (12)
Australia	2 (1)	0	2 (1)
Africa	5 (4)	0	6 (3)
Post-training Practice Years	*n* = 143	*n* = 23	*n* = 176
0–5 years	38 (27)	1 (4)	39 (22)
6–10 years	19 (13)	5 (22)	26 (15)
11–15 years	20 (14)	6 (26)	29 (17)
16–20 years	18 (13)	3 (13)	23 (13)
20 + years	48 (34)	8 (35)	59 (34)
Institution	*n* = 143	*n* = 23	*n* = 176
Community-based non-teaching hospital	6 (4)	3 (13)	12 (7)
Non-university teaching hospital	20 (14)	1 (4)	22 (12)
Academic teaching hospital	117 (82)	19 (83)	142 (81)
Annual PDAC Patient Size-Individual: In a typical year, how many patients with PDAC do you manage?^b^	*n* = 143	*n* = 23	*n* = 176
0–10	8 (6)	13 (56)	26 (15)
11–25	23 (16)	5 (22)	30 (17)
26–50	29 (20)	2 (9)	32 (18)
51–100	45 (31)	2 (9)	49 (28)
100+	38 (27)	1 (4)	39 (22)
Annual PDAC Patient Size-Institution: In a typical year, how many patients with PDAC are seen at your institution?^b^	*n* = 143	*n* = 23	*n* = 176
0–25	8 (6)	5 (22)	20 (11)
26–50	19 (13)	8 (35)	28 (16)
51–100	30 (21)	6 (26)	38 (22)
101–200	33 (23)	1 (4)	34 (19)
200+	53 (37)	3 (13)	56 (32)
Is the treatment plan for patients with PDAC routinely discussed by a tumor board?	*n* = 143	*n* = 23	*n* = 176
Yes	133 (93)	22 (96)	164 (94)
No	10 (7)	1 (4)	11 (6)
Who typically attends your tumor board?^c^	*n* = 132	*n* = 21	*n* = 159
Surgery	132 (100)	21 (100)	156 (98)
Medical Oncology	130 (99)	21 (100)	156 (98)
Radiation Oncology	104 (79)	21 (100)	131 (82)
Gastroenterology	89 (67)	15 (71)	107 (67)
Pathology	92 (70)	19 (91)	113 (71)
Radiology	124 (94)	19 (91)	145 (91)
Palliative care or other supportive care	26 (20)	6 (29)	34 (21)
Nutrition	26 (20)	3 (14)	31 (20)
Does your institution offer neoadjuvant or adjuvant radiotherapy for PDAC?	*n* = 142	*n* = 22	*n* = 170
Yes	124 (87)	21 (96)	151 (89)
No	18 (13)	1 (5)	19 (11)
For patients recommended to receive radiotherapy, where do most (> 50%) of your patients receive their radiotherapy?	*n* = 142	*n* = 22	*n* = 170
At your center	108 (76)	20 (91)	134 (79)
At a different center	34 (24)	2 (9)	36 (21)

^a^ Total cohort includes surgeons, radiation oncologists, 2 medical oncologists, and 8 “other” specialists

^b^ Comparisons across groups show statistically significant differences, *p* < 0.001

^c^ Check-all-that-apply questions: the percentages do not add up to 100

*PDAC* pancreatic ductal adenocarcinoma

### Perspectives of Neoadjuvant Radiotherapy Utilization

For non-metastatic PDAC patients, there was wide variation regarding indications for neoadjuvant RT. Nearly one-fifth of the entire cohort (*n* = 32, 19%) did not believe there was any indication for neoadjuvant RT and/or did not offer it to their patients (Table 2). Among those respondents, the majority were surgeons (*n* = 27, 84%) as opposed to radiation oncologists (*n* = 5, 23%). The most commonly cited indications for neoadjuvant RT in non-metastatic PDAC patients were locally advanced (*n* = 88, 52%) and surgically unresectable (*n* = 86, 51%) disease, local tumor progression on chemotherapy (*n* = 79, 47%), and arterial abutment of the primary tumor (*n* = 68, 40%), however this also differed by specialty.

**Table 2:** Management preferences for neoadjuvant radiotherapy and radiotherapy delivery

Resectablility classification	Surgeons	Radiation oncologists		Total cohort^a^
	*N* (%)	*N* (%)		*N* (%)
All Non-metastatic Patients				
What do you consider to be indications for neoadjuvant RT for PDAC? ^c^	*n* = 142	*n* = 22		*n* = 170
No Indication/Do not offer	27 (19)	5 (23)		32 (19)
All non-metastatic PDAC patients should receive neoadjuvant radiotherapy	8 (6)	1 (5)		12 (7)
Enlarged local lymphadenopathy	13 (9)	3 (14)		18 (11)
Abutment with the SMV/PV	30 (21)	8 (36)		39 (23)
Encasement with SMV/PV amenable to reconstruction	47 (33)	10 (46)		59 (35)
Abutment with CA, SMA, or CHA	59 (42)	7 (32)		68 (40)
Locally Advanced PDAC	72 (51)	12 (55)		88 (52)
Surgically Unresectable PDAC	69 (49)	13 (59)		86 (51)
Local tumor progression on chemotherapy	66 (47)	9 (41)		79 (47)
Pain symptoms	51 (36)	10 (46)		62 (37)
Resectable				
For patients with resectable PDAC, how do you integrate neoadjuvant RT?	*n* = 141	*n* = 22		*n* = 169
We do not offer neoadjuvant radiotherapy for patients with resectable PDAC	115(82)	15(68)		132(78)
Before induction systemic therapy	2(1)	0		4(2)
After some period of induction systemic therapy	23(16)	6(27)		31(18)
As the only form of neoadjuvant therapy	1(1)	1(5)		2(3)
What estimated percentage of resectable PDAC patients that you manage receive neoadjuvant RT?	*n* = 26	*n* = 7		*n* = 37
0–20%	11 (42)	6 (86)		19 (51)
20–40%	5 (19)	1 (14)		8 (22)
40–60%	3 (12)	0		3 (8)
60–80%	5 (19)	0		5 (14)
> 80%	2 (8)	0		2 (5)
What types of neoadjuvant RT do you offer patients with resectable PDAC?	*n* = 26	*n* = 7		*n* = 37
SBRT (stereotactic body radiation therapy)	6(23)	1(14)		8(22)
Chemoradiation with conventional dose/fractionation	10(39)	3(43)		15(40)
Chemoradiation with dose-escalated IMRT (intensity-modulated radiation therapy)	3(12)	3(43)		7(19)
Unknown – defer type to the Radiation Oncologist	5(19)	0		5(14)
Other	2(7)	0		2(5)
Borderline Resectable Pancreatic Cancer				
For patients with BRPC, how do you integrate neoadjuvant RT?				
We do not offer neoadjuvant radiotherapy for patients with resectable PDAC	75 (54)	7 (35)		84 (50)
Before induction systemic therapy	3 (2)	0		5 (3)
After some period of induction systemic therapy	59 (42)	12 (60)		73 (44)
As the only form of neoadjuvant therapy	3 (2)	1 (5)		4 (3)
What estimated percentage of BRPC patients that you manage receive neoadjuvant RT?	*n* = 64	*n* = 12		*n* = 79
0–25%	26 (41)	5 (42)		33 (42)
25–50%	13 (20)	3 (25)		17 (22)
50–75%	9 (14)	2 (17)		11 (14)
> 75%	16 (25)	2 (17)		18 (23)
What types of neoadjuvant RT do you offer patients with BRPC?	*n* = 64	*n* = 12		*n* = 79
SBRT (stereotactic body radiation therapy)	18 (28)	4 (33)		22 (28)
Chemoradiation with conventional dose/fractionation	20 (31)	5 (42)		25 (31)
Chemoradiation with dose-escalated IMRT (intensity-modulated radiation therapy)	12 (19)	3 (25)		18 (23)
Unknown – defer type to the Radiation Oncologist	11 (17)	0		11 (14)
Other	3 (5)	0		3 (4)
Locally Advanced Pancreatic Cancer				
For patients with LAPC, how do you integrate neoadjuvant RT?	*n* = 94	*n* = 12		*n* = 112
We do not offer radiotherapy for patients with locally advanced PDAC	42 (30)	4 (22)		47 (29)
Before induction systemic therapy	2 (2)	0		4 (2)
After some period of induction systemic therapy	95 (68)	14 (78)		111 (69)
As the only form of neoadjuvant therapy	0	0		0
For patients with LAPC, how many months of chemotherapy are ideally given prior to transitioning to RT?	*n* = 94	*n* = 14		*n* = 112
1–3 months	17 (18)	3 (21)		22 (20)
4–6 months	57 (61)	10 (71)		68 (61)
More than 6 months	15 (16)	1 (7)		16 (14)
We don’t offer radiation therapy for locally advanced PDAC	1 (1)	0		1 (1)
Other	4 (4)	0		5 (5)
What estimated percentage of LAPC patients that you manage receive neoadjuvant RT?	*n* = 135	*n* = 18		*n* = 158
0–25%	83 (62)	12 (67)		97 (61)
25–50%	14 (10)	3 (17)		19 (12)
50–75%	11 (8)	1 (6)		12 (8)
> 75%	27 (20)	2 (11)		30 (19)
What types of neoadjuvant RT do you offer patients with LAPC?	*n* = 135	*n* = 18		*n* = 158
SBRT (stereotactic body radiation therapy)	40 (30)	4 (22)		45 (29)
Chemoradiation with conventional dose/fractionation	30 (22)	5 (28)		37 (23)
Chemoradiation with dose-escalated IMRT (intensity-modulated radiation therapy)	16 (12)	5 (28)		23 (15)
Unknown – defer type to the Radiation Oncologist	37 (27)	1 (5)		38 (24)
Other	12 (9)	3 (17)		15 (9)
Radiotherapy Delivery				
Which chemotherapy do you use as a radiosensitizer?^c^	*n* = 132	*n* = 18	*n* = 55	
Gemcitabine	72 (55)	10 (56)	85 (55)	
5-Fluorouracil	61 (46)	12 (67)	77 (50)	
Other	29 (22)	3 (17)	33 (21)	
At your institution, what is the preferred timing for surgical resection following neoadjuvant radiotherapy?	*n* = 132	*n* = 18	*n* = 55	
1–2 weeks	4 (3)	1 (6)	5 (3)	
3–4 weeks	27 (21)	5 (28)	34 (22)	
5–6 weeks	47 (36)	5 (28)	54 (35)	
> 6 weeks	27 (21)	4 (22)	31 (20)	
Depends on the type of radiation	12 (9)	1 (6)	14 (9)	
Other	15 (11)	2 (11)	17 (11)	
What type of delivery system do you generally utilize for treating pancreatic cancer patients?^c^	*n* = 132	*n* = 18	*n* = 55	
Traditional LINAC	61 (46)	13 (72)	76 (49)	
MR-guided LINAC	20 (15)	3 (17)	24 (16)	
CyberKnife	15 (11)	2 (11)	19 (12)	
Unknown	49 (37)	1 (6)	50 (32)	
Other	8 (6)	1 (6)	10 (7)	
In addition to the gross disease, what do you include in your target volume for patients receiving neoadjuvant radiation therapy?^c, d^	*n* = 0	*n* = 18	*n* = 18	
Elective lymph nodes	0	6 (33)	6 (33)	
Triangle volume (space between celiac artery, superior mesenteric artery, common hepatic artery, and portal vein.	0	6 (33)	6 (33)	
No elective targeting.	0	7 (39)	7 (39)	
Other: Free Text	0	3 (17)	3 (17)	
In addition to the gross disease, what do you include in your target volume for patients receiving definitive radiation therapy?^c, d^	*n* = 0	*n* = 18	*n* = 18	
Elective lymph nodes	0	7 (39)	7 (39)	
Triangle volume (space between celiac artery, superior mesenteric artery, common hepatic artery, and portal vein.	0	9 (50)	9 (50)	
No elective targeting.	0	5 (28)	5 (28)	
Other: Free Text	0	3 (17)	3 (17)	
In your opinion, does neoadjuvant radiotherapy result a technically more challenging surgical resection?^e^	*n* = 132	*n* = 0	*n* = 132	
Yes, always	28 (21)	0	28 (21)	
Yes, sometimes	65 (49)	0	65 (49)	
No, it does not impact the surgical field or the technical difficulty of a surgical resection	39 (30)	0	39 (30)	
How does neoadjuvant radiotherapy impact your decision to offer a minimally-invasive approach to pancreatic resections?^e^	*n* = 130	*n* = 0	*n* = 130	
Neoadjuvant radiotherapy has no impact on my decision to offer a minimally-invasive approach to pancreatic resection	41 (31)	0	41 (31)	
I am hesitant to offer a minimally-invasive approach to pancreatic resection for patients who receive neoadjuvant radiotherapy	57 (44)	0	57 (44)	
Not Applicable/I do not perform minimally-invasive pancreatic resections	32 (25)	0	32 (25)	

^a^ Total cohort includes surgeons, radiation oncologists, 2 medical oncologists, and 8 “other” specialists

^b^Comparisons across groups show statistically significant differences, *p* < 0.001

^c^ Check-all-that-apply questions: the percentages do not add up to 100

^d^ Radiation oncologist only question

^e^ Surgeon only question

*RT* radiotherapy, *SBRT* stereotactic body radiotherapy, *IMRT* intensity modulated radiotherapy, *SMA* superior mesenteric artery, *PV* portal vein, *CA* celiac axis, *CHA* common hepatic artery, *LAPC* locally advanced pancreatic cancer, *BRPC* borderline resectable pancreatic cancer, *PDAC* pancreatic ductal adenocarcinoma

### Clinical Impact of Neoadjuvant Radiotherapy

**Fig. 1:**
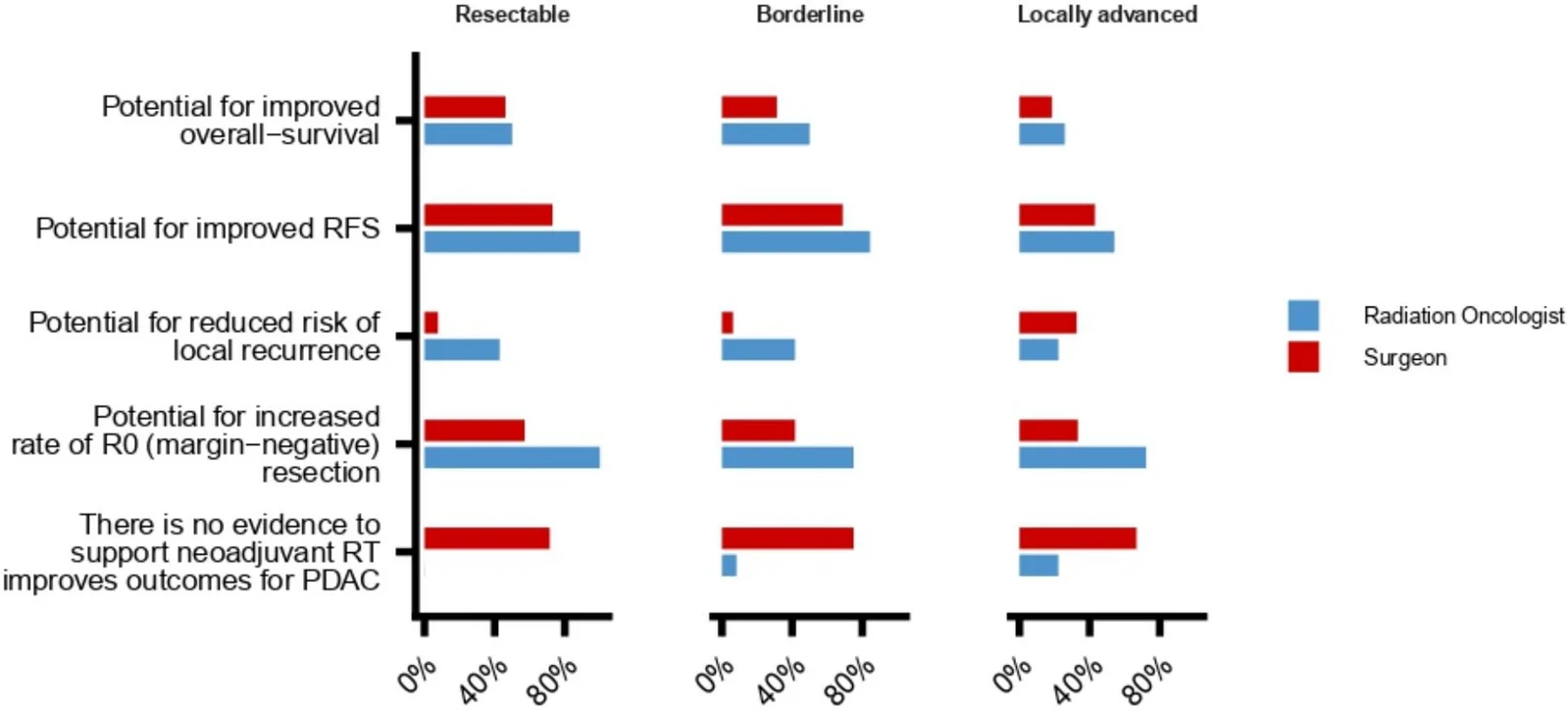
Surgeon and radiation oncologist perceptions of effectiveness for neoadjuvant radiotherapy across resectability classifications (RFS: recurrence free survival; RT: radiotherapy; PDAC: Pancreatic Ductal Adenocarcinoma)

Among respondents who offer neoadjuvant RT, perceptions of its effectiveness varied by resectability classification (Fig. 1). For resectable PDAC, 89% of surgeons (*n* = 23) and 71% of radiation oncologists (*n* = 5) believe neoadjuvant RT had potential to increase rate of R0 margin-negative resection, though fewer perceived a potential for improved overall survival (surgeons: 46%, *n* = 12; radiation oncologists: 43%, *n* = 3). For BRPC, the perceived potential for R0 resection remained high (surgeons: 84%, *n* = 54; radiation oncologists: 75%, *n* = 9), while perceptions of benefit for LAPC were diminished, with 33% of surgeons (*n* = 44) and 22% of radiation oncologists (*n* = 4) reporting no evidence to support neoadjuvant RT for LAPC outcomes.

### Perceptions of Radiotherapy by Resectability Classification

Opinions for neoadjuvant RT indications differed according to resectability classification (resectable, BRPC, LAPC) and specialty (Table 2). The overwhelming majority of surgeons responded that they do not offer neoadjuvant RT for patients with resectable disease (*n* = 115, 82%) compared to 68% of radiation oncologists (*n* = 15). Notably, this reported low utilization in resectable disease contrasted with the high proportion of respondents who believed neoadjuvant RT could improve R0 resection in this setting (Fig. 1). Perspectives for patients with BRPC (Surgeons: *n* = 59, 42% vs. Radiation oncologists: *n* = 12, 60%) and LAPC (Surgeons: *n* = 95, 68% vs. Radiation oncologists: *n* = 14, 78%) were more consistent between surgeons and radiation oncologists, with most integrating its use after induction chemotherapy (Table 2). Most respondents indicated a period of 4–6 months as the ideal time of induction chemotherapy prior to transitioning to neoadjuvant RT for patients with LAPC (*n* = 68, 61%).

### Radiotherapy Delivery Preferences

Among respondents who offer neoadjuvant RT, the most commonly used radiosensitizing agents were gemcitabine (*n* = 85, 55%) and 5-fluorouracil (*n* = 77, 50%). There was wide variation in the preferred timing for surgical resection: 5–6 weeks (*n* = 54, 35%) was most preferred, followed by 3–4 weeks (*n* = 34, 22%) and > 6 weeks (*n* = 31, 20%). The most commonly used delivery system was a traditional linear accelerator (LINAC) (*n* = 76, 49%), though a substantial proportion of surgeons (37%, *n* = 49) were unaware of the delivery system used at their institution (Table 2). MR-guided LINAC (*n* = 24, 16%) and CyberKnife (*n* = 19, 12%) were less frequently utilized. Among radiation oncologists, target volumes for neoadjuvant RT varied, with 39% (*n* = 7) employing no elective targeting beyond gross disease, 33% (*n* = 6) including elective lymph nodes, and 33% (*n* = 6) targeting the celiac-SMA-portal vein triangle volume.

### Radiotherapy in the Adjuvant Setting

Regarding adjuvant RT, nearly one-third of respondents (*n* = 46, 30%) did not consider any indication for adjuvant RT for resected PDAC, with a higher proportion of surgeons (32%, *n* = 42) expressing this view (Table 3). The most commonly cited indication for adjuvant RT was a positive margin (*n* = 97, 63%), followed by positive nodal disease (*n* = 46, 30%) and perineural or lymphovascular invasion (*n* = 37, 24%). Radiation oncologists were more likely than surgeons to endorse adjuvant RT for positive nodal disease (56% vs. 24%) and perineural/lymphovascular invasion (33% vs. 20%). Among those offering adjuvant RT, the majority (*n* = 57, 54%) preferred to administer it following completion of adjuvant chemotherapy.

**Table 3:** Perceptions and preferences for use of radiotherapy in the adjuvant and metastatic settings

Respondent perceptions and preferences	Surgeons	Radiation oncologists	Total cohort^a^
	*N* (%)	*N* (%)	*N* (%)
Metastatic Setting			
Would you consider radiotherapy in the following scenarios? ^b^	*n* = 128	*n* = 16	*n* = 149
Definitive local therapy for unresectable localized disease without metastases combined with systemic chemotherapy	97 (76)	14 (88)	113 (76)
Definitive local therapy for local disease progression without metastases combined with systemic chemotherapy	80 (63)	12 (75)	93 (62)
Definitive local therapy for unresectable localized disease without metastases in a patient unable to tolerate chemotherapy or with declining functional status	86 (67)	14 (88)	103 (69)
Definitive local therapy for local disease progression in the presence of metastases combined with systemic chemotherapy	17 (13)	5 (31)	25 (17)
Bleeding from Primary Tumor Site	71 (56)	12 (75)	85 (57)
Pain or other symptomology related to the primary tumor	91 (71)	15 (94)	107 (72)
Treatment for metastatic sites (e.g., Liver, Lung, or Bone) in patients with oligometastatic PDAC	59 (46)	11 (69)	72 (48)
Do you use SBRT as a treatment for metastatic sites in patients with oligometastatic PDAC?	*n* = 58	*n* = 11	*n* = 71
Yes	53 (91)	11 (100)	66 (93)
No	5 (9)	0	5 (7)
In what instances do you consider SBRT treatment of metastatic sites for the management of oligometastatic PDAC? ^b^	*n* = 58	*n* = 11	*n* = 71
Liver-only disease	36 (62)	4 (36)	40 (56)
Lung-only disease	32 (55)	5 (46)	37 (52)
Regional or distant lymph node recurrence only	15 (26)	4 (36)	20 (28)
Stability on systemic therapy regardless of metastatic site	25 (43)	10 (91)	37 (52)
Other	11 (19)	0	11 (16)
What benefit do you believe SBRT to metastatic sites has in the setting of oligometastatic PDAC?	*n* = 58	*n* = 11	*n* = 71
None	4 (7)	0	4 (6)
Disease-free survival benefit	17 (29)	4 (36)	21 (30)
Overall survival benefit	13 (22)	3 (27)	17 (24)
Symptomatic benefit	18 (31)	4 (36)	23 (32)
Other	6 (10)	0	6 (8)
Adjuvant Therapy			
What do you consider to be indications for adjuvant radiotherapy for patients with resected PDAC?^b^	*n* = 130	*n* = 18	*n* = 153
No Indication/Do not offer	42 (32)	4 (22)	46 (30)
All Resected tumors	5 (4)	2 (11)	9 (6)
Positive Surgical Margin	79 (61)	13 (72)	97 (63)
Positive Nodal Disease	31 (24)	10 (56)	46 (30)
Negative Nodal Disease	6 (5)	2 (11)	9 (6)
Perineural or lymphovascular invasion	26 (20)	6 (33)	37 (24)
Other pathologic or clinical features	12 (9)	2 (11)	14 (9)
Other: Free text	9 (7)	0	9 (6)
How long after surgery do you recommend to start adjuvant radiotherapy for patients with resected PDAC?	*n* = 88	*n* = 13	*n* = 106
4–8 weeks prior to starting adjuvant systemic therapy	22 (25)	3 (23)	27 (26)
> 8 weeks prior to starting adjuvant systemic therapy	10 (11)	3 (23)	15 (14)
Following completion of adjuvant systemic therapy	50 (57)	6 (46)	57 (54)
Not applicable/I do not offer adjuvant radiotherapy	6 (7)	1 (8)	7 (7)

^a^ Total cohort includes surgeons, radiation oncologists, 2 medical oncologists, and 8 “other” specialists

^b^ Check-all-that-apply questions: the percentages do not add up to 100

*SBRT* stereotactic body radiotherapy, *PDAC* pancreatic ductal adenocarcinoma

### Impact of Neoadjuvant Radiotherapy on the Surgical Field

**Fig. 2:**
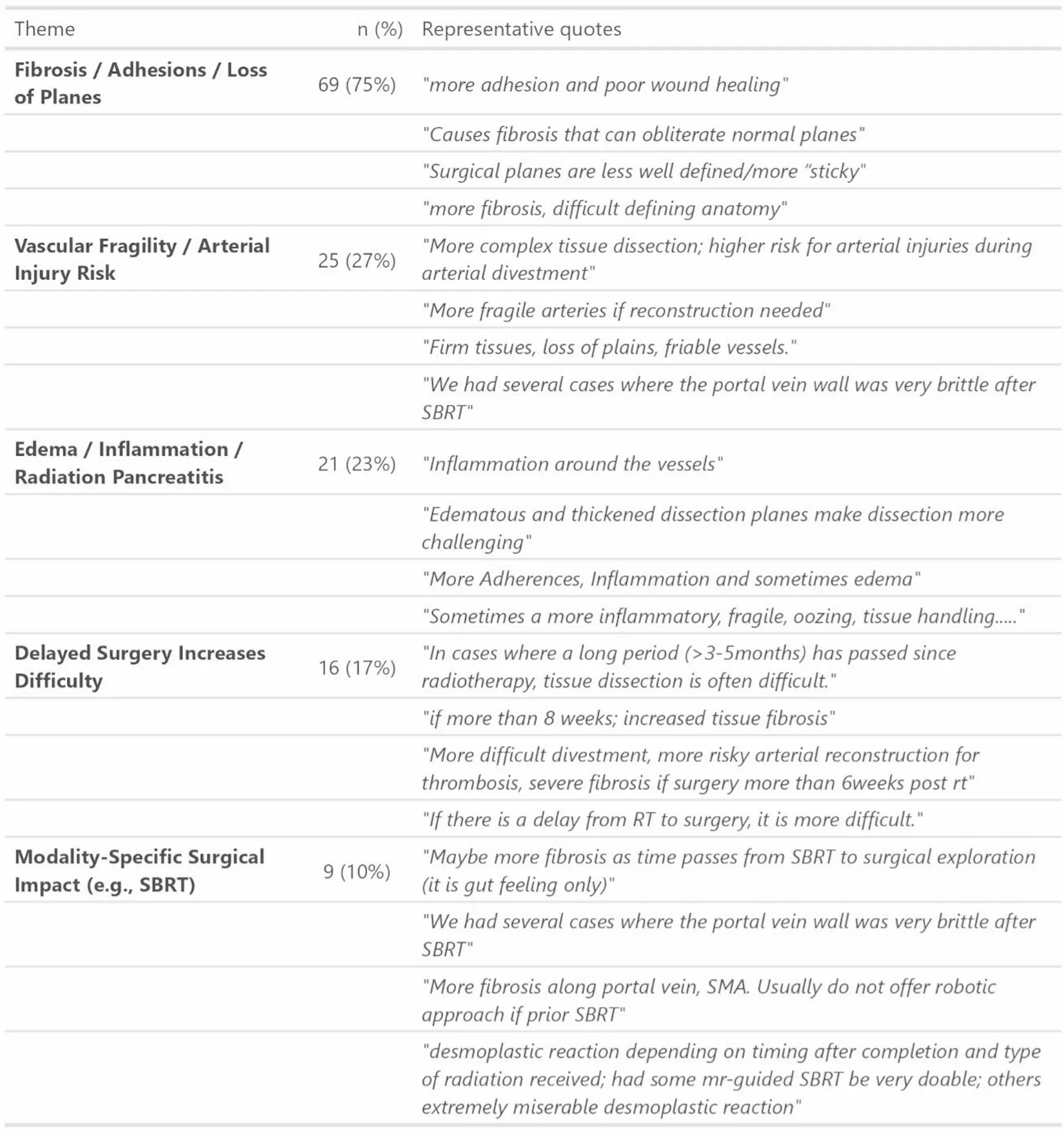
Surgeon perceptions of the impact of radiotherapy on surgical resection (RT: radiotherapy; SBRT: stereotactic body radiotherapy; MRI: magnetic resonance imaging; SMA: superior mesenteric artery)

The majority of surgeons (*n* = 93, 70%) believe neoadjuvant RT results in a more challenging technical resection, with 21% (*n* = 28) stating it always increases difficulty and 49% (*n* = 65) reporting it sometimes does (Table 2). Regarding minimally-invasive approaches, 44% of surgeons (*n* = 57) reported hesitancy to offer a minimally-invasive resection following neoadjuvant RT, while 32% (*n* = 41) stated it had no impact on their approach. Among the 92 surgeons who provided qualitative responses describing the impact of neoadjuvant RT (Fig. 2), the most commonly cited themes included fibrosis, adhesions, and loss of tissue planes (*n* = 69, 75%), vascular fragility and increased arterial injury risk (*n* = 25, 27%), edema and radiation-induced inflammation (*n* = 21, 23%), and increased difficulty with delayed surgery beyond 6–8 weeks (*n* = 16, 17%). Several respondents noted modality-specific differences in surgical impact, with SBRT cited as occasionally resulting in more severe fibrosis and vascular changes (*n* = 9, 10%).

### Radiotherapy in the Metastatic Setting

Perceptions on the use of RT in metastatic PDAC varied widely (Table 3). For instance, the majority of surgeons (*n* = 91, 71%) and radiation oncologists (*n* = 15, 94%) would consider RT for pain or other symptomatology related to the primary tumor. However, only 91% of surgeons (*n* = 53) believe radiotherapy is indicated for the treatment of metastatic sites in patients with oligometastatic PDAC, compared to 100% (*n* = 11) of radiation oncologists. Furthermore, the management of oligometastatic disease varied greatly based on site of disease with 56% (*n* = 40) of respondents considering its use in liver-only disease versus 28% (*n* = 20) considering its use with regional or distant lymph node recurrence.

## Discussion

In this international survey of 176 surgeons, radiation oncologists, and other PDAC specialists from six continents, we identified substantial variation in perceptions of effectiveness, management preferences, and the propensity to utilize RT for PDAC. Nearly one in five respondents did not consider any indication for neoadjuvant RT, while almost one-third did not endorse adjuvant RT for any patient with resected disease. When neoadjuvant RT is offered, its use varies considerably by resectability classification, with greater consensus reported for BRPC and LAPC compared to resectable and metastatic PDAC.

The wide variation in attitudes and preferences observed in this study likely reflects inconsistencies in consensus guidelines and the mixed results of available evidence. In the adjuvant setting, historical studies were mixed with respect to the role of adjuvant radiation/chemoradiation, with the GITSG 9173 study demonstrating improved outcomes, but with the EORTC 40,891 and ESPAC-1 studies reporting no benefit [1, 3, 14]. More recently, the RTOG 0848 randomized trial did not meet its primary endpoint, as the addition of adjuvant chemoradiation following adjuvant chemotherapy did not significantly improve overall survival in the overall study population compared to adjuvant chemotherapy alone among patients with resected pancreatic head cancer. However, a treatment-by-nodal-status analysis found that chemoradiation improved both overall survival and disease-free survival specifically in node-negative patients [15]. It is interesting that despite the RTOG 0848 findings, pathologic lymph node involvement was often evoked as an indication for adjuvant RT, particularly among radiation oncologists compared to surgeons. This divergence likely reflects differing interpretations of nodal involvement as a marker of locoregional versus systemic disease. Among most solid organ malignancies, nodal involvement may be interpreted either as evidence of systemic dissemination, favoring systemic therapy, or as locoregional disease that remains amenable to local ablative therapies control. This distinction is particularly relevant given the RTOG 0848 findings among node-negative patients [15]. Our findings therefore suggest a possible mismatch between contemporary evidence and reported practice, reinforcing the need for clearer consensus regarding patient selection for adjuvant RT. Certainly, more data is required to clarify which patients benefit most from adjuvant RT, as current practice patterns are clearly mixed and may be incongruent with true drivers of locoregional recurrence. Indeed, a body of literature, now supported by RTOG 0848, suggests that neural tract spread, as opposed to lymphatic spread, is a more important driver of locoregional recurrence [16–18].

In the pre-operative setting, the PREOPANC trial demonstrated long-term overall survival benefit, driven by a significant improvement in locoregional control, in patients with resectable or BRPC treated with pre-operative chemoradiation compared to upfront surgery [5]. However, the pre-operative chemoradiation arm did not include modern, multi-agent systemic therapy. As such, the pre-operative chemoradiation arm from PREOPANC was subsequently compared to neoadjuvant mFOLFIRINIOX in PREOPANC-2, which did not result in a difference in survival outcomes [19]. As a result, a more relevant question to modern practice is the possible additive benefit that pre-operative radiation may offer beyond neoadjuvant multi-agent chemotherapy. While the A021501 trial evaluated this question, the poor outcomes in the hypofractionated radiation arm, which contrast with outcomes from high-volume centers, raise concerns about its applicability to modern practice [4, 20]. In the setting of LAPC without resection, the LAP-07 study explored the role of consolidative chemoradiation following upfront chemotherapy and did not find an survival benefit [6]. However, local PFS was improved, and more importantly, the study employed single agent chemotherapy and low radiation dose. The importance of dose-escalation for achieving radiation response with PDAC has since been demonstrated, and the NRG GI011 trial is now underway to reassess the role of consolidative “ablative” radiation in the unresectable LAPC cohort [21, 22]. In addition, the recently published CONKO-007 demonstrated significant long-term locoregional control with neoadjuvant radiation in LAPC patients that achieved resection, with signal of potential long-term survival benefit as well [7].

Our findings identified a notable disconnect between perceived efficacy and reported utilization of neoadjuvant RT, particularly in resectable PDAC. Among respondents who offer neoadjuvant RT, confidence in its ability to improve R0 resection was highest in resectable disease, with 89% of surgeons and 71% of radiation oncologists endorsing this potential (Fig. 1). Yet, the majority of surgeons (82%) and radiation oncologists (68%) reported that they do not offer neoadjuvant RT to patients with resectable disease. This apparent discrepancy between perceived oncologic benefit and actual clinical use is informative and identifies an area of exploration for future studies. Although surgeons perceived a potential RT benefit for R0 resection in resectable disease, far fewer believed neoadjuvant RT improved overall survival (46% of surgeons and 43% of radiation oncologists), suggesting that a perceived margin benefit alone may be insufficient to justify its use when upfront resection is already achievable. In this setting, clinicians may perceive limited incremental benefit over modern multi-agent systemic therapy and may be reluctant to risk overtreatment, added toxicity, increased operative difficulty, or delays to surgery in otherwise resectable patients. Competing concerns regarding early systemic progression, along with existing guideline recommendations that reserve RT for borderline resectable and locally advanced disease, may further explain why the perceived biological benefit of RT does not translate into increased use.

Not surprisingly, the findings of this survey also reveal important differences in attitudes toward radiotherapy between surgeons and radiation oncologists, suggesting that specialty-specific training and experiences influence treatment recommendations in the absence of definitive evidence. This phenomenon of “specialty bias” has previously been demonstrated across multiple decades and numerous disease sites [14, 23–26]. While radiation oncologists were consistently more likely to endorse neoadjuvant RT across all clinical situations, a large majority of surgeons (70%) endorsed beliefs that RT increases the technical difficulty of resection. Qualitative responses suggest that concerns regarding fibrosis, loss of tissue planes, vascular fragility, and increased inflammation were consistent surgical perceptions. These findings have important implications for the multidisciplinary management of PDAC, as surgical concerns regarding tissue quality and dissection difficulty may influence referral patterns and willingness to recommend neoadjuvant RT. For example, nearly 44% of surgeons expressed hesitancy to offer minimally-invasive resection following neoadjuvant RT, which may have downstream implications for patient recovery and perioperative outcomes [27, 28]. The impact of neoadjuvant RT on technical difficulty during resection is an area that warrants further investigation, particularly as newer modalities such as MR-guided LINAC and SBRT may have differential effects on surrounding tissue. These reported perceptions of surgeons on the impact of RT on the surgical field presents an opportunity for future studies to objectively measure the impact of RT on operative difficulty and outcomes related to this. Future studies should objectively evaluate operative difficulty, vascular reconstruction, conversion from minimally-invasive to open approaches, blood loss, and perioperative outcomes following different neoadjuvant RT modalities, to determine whether these widely held surgical perceptions correspond to measurable operative effects. Regardless, these results highlight the extreme importance of nuanced multidisciplinary discussion for every patient with PDAC, with representation from all relevant oncologic sub-specialties.

This survey also revealed variation in the technical aspects of RT delivery that may contribute to heterogeneity in utilization and perceived benefits across institutions. While the traditional LINAC remains the most commonly utilized platform, a substantial proportion of surgeons were unaware of the delivery system nor the fractionation preference used at their institution, highlighting a potential gap in multidisciplinary communication. However, even among radiation oncologists, there was no consensus regarding target volumes, with roughly equal proportions endorsing elective lymph node targeting, triangle volume targeting, and gross disease-only approaches alike. Certainly, target volume delineation has been highly variable across key randomized trials to-date. However, there has been growing consensus that elective targeting, likely informed by neural tract anatomy, is key for achieving optimal locoregional control [18, 29]. Similarly, the use of RT in the metastatic setting varied widely, though both surgeons and radiation oncologists agreed on the role of palliative RT for symptom management. The growing interest in SBRT for oligometastatic disease, with 93% of respondents who treat oligometastatic PDAC reporting use of SBRT, suggests an evolving paradigm that will require additional prospective evidence beyond recently published EXTEND trial to define optimal patient selection and treatment parameters [30]. These and other areas with low quality evidence should be the focus of further multi-institutional investigation to reduce the wide variation of practices seen internationally.

This study has numerous limitations. First, the sample size of this survey is small (*n* = 176), and though unmeasured, the survey response rate is likely low given the high membership of the email listservs utilized. Nevertheless, these findings are not unexpected for an external email survey without incentive, and are comparable to previously published surveys of similar scope and purpose [12, 13, 31, 32]. Second, as with any anonymous survey, responses are self-reported, cannot be verified for accuracy, and represent respondent attitudes at one point in time, but may not represent actual treatment patterns. Third, survey responses are subject to selection bias among those choosing to respond. Considering most respondents to this survey were surgeons, these results may not be generalizable to nonresponding surgeons’ or radiation oncologists’ attitudes and preferences. Furthermore, the near absence of responses from medical oncologists is an important limitation given the central role of medical oncologists in coordinating systemic therapy and its sequencing with RT. However, considering that most patients at respondent institutions are discussed at a multidisciplinary conference, and multimodality treatment recommendations are often determined by these multidisciplinary discussions, it is reasonable to believe these results represent current attitudes at respondent institutions. Similarly, these survey responses may not be generalizable to regions outside of North America and Europe. Regardless, an electronic survey of this nature was the only feasible method to conduct this research, and these survey results are comparable to prior reports.

## Conclusions

In summary, this international survey suggests substantial variation in surgeon and radiation oncologist perceptions of effectiveness, management preferences, and propensity to offer RT for patients with PDAC. However, given the limitations in response rate as well as the over-representation of surgeons, interpretation of these findings may not generalize to the broader population and global specialty-specific practice patterns cannot be established from this study.

Despite four decades of research, there remains a lack of high-quality evidence to support consistent recommendations for the use of neoadjuvant and adjuvant RT across resectability classifications. The heterogeneity in practice reported in this study, coupled with specialty-specific differences in attitudes, underscores the importance of ongoing and future multi-institutional trials evaluating modern RT techniques and their role in multidisciplinary management of PDAC. Until stronger evidence is available, treatment decisions regarding RT should be individualized through robust and nuanced multidisciplinary discussions that incorporate the perspectives of all oncologic sub-specialists. Going forward, consensus guidelines from major oncologic societies should seek to harmonize recommendations supported by weak evidence, to reduce unwarranted variation and potentially improve outcomes [33, 34].

## Data Availability

No datasets were generated or analysed during the current study.
